# Dual regulation of osteoclastogenesis and osteogenesis for osteoporosis therapy by iron oxide hydroxyapatite core/shell nanocomposites

**DOI:** 10.1093/rb/rbab027

**Published:** 2021-08-23

**Authors:** Mengye Li, Shengxiang Fu, Zhongyuan Cai, Danyang Li, Li Liu, Di Deng, Rongrong Jin, Hua Ai

**Affiliations:** National Engineering Research Center for Biomaterials, Sichuan University, Chengdu 610064, China; National Engineering Research Center for Biomaterials, Sichuan University, Chengdu 610064, China; National Engineering Research Center for Biomaterials, Sichuan University, Chengdu 610064, China; Scientific Research Center, The Seventh Affiliated Hospital, Sun Yat-sen University, Shenzhen 518107, China; National Engineering Research Center for Biomaterials, Sichuan University, Chengdu 610064, China; National Engineering Research Center for Biomaterials, Sichuan University, Chengdu 610064, China; National Engineering Research Center for Biomaterials, Sichuan University, Chengdu 610064, China; National Engineering Research Center for Biomaterials, Sichuan University, Chengdu 610064, China; Department of Radiology, West China Hospital, Sichuan University, Chengdu 610041, China

**Keywords:** hydroxyapatite, superparamagnetic iron oxide, osteoporosis treatment, nanocomposites

## Abstract

Osteoporosis is a skeletal disorder resulted in significant structural and functional changes, arousing a wide concern for the high prevalence and cost. Imbalance between osteoclastogenesis and osteogenesis have been verified as a main pathology etiology and considered an efficient therapy target in both clinical and pre-clinical studies. In recent years, inorganic nanomaterials have shown provable activities on osteoclastogenesis inhibition and osteogenesis promotion, respectively. Hence, in this study, a class of hydroxyapatite coated superparamagnetic iron oxide nanoparticles (SPIO@HA) were developed with a core−shell structure for targeting both osteoclastogenesis and osteogenesis. The optimal ratio of SPIO@15HA (Fe/Ca = 1:15, mol/mol) was screened to obtain dual function for inducing both bone formation and preventing bone resorption. The obtained nanocomposites significantly prevented the bone loss of ovariectomized (OVX) mice and increased bone mineral density (BMD) by 9.4%, exhibiting high bone accumulation in magnetic resonance imaging evaluation and reasonable biosafety profile. The mechanism study revealed that SPIO@15HA can suppress bone marrow monocyte derived osteoclast differentiation through TRAF6−p62−CYLD signaling complex regulation. Meanwhile, it could activate MSC osteogenic differentiation by TGF-β, PI3K-AKT and calcium signaling pathway regulation. Moreover, incubation of SPIO@15HA with MSC resulted in several cytokines overexpression such as osteoprotegerin (OPG), CSF2, CCL2 etc., which are responsible for maintaining the bone remodeling balance. The dual function of as-prepared SPIO@15HA may find a new way for designing of inorganic components containing core/shell nanomaterials for osteoporosis treatment.

## Introduction

Osteoporosis, characterized by diminished bone mass and strength, is a well-known intractable skeletal disorder that leads to increased fracture risk and enormous health care costs, mainly caused by aging, chronic disease (rheumatoid arthritis, long-term kidney disease, diabetes) and medication side-effects [1, 2]. It was investigated that the proportion of fractures caused by osteoporosis or low bone mass ranged from 20% to 30% in countries worldwide [3]. Therefore, identifying effective new therapeutic paradigms is of critical importance. Osteoporosis results from disturbed balance in the bone remodeling process composed of osteoclast-mediated bone resorption and osteoblast-mediated bone formation [4, 5]. Thus, clinically approved drugs for osteoporosis treatment are specific of osteoblastic or osteoclastic activities alternatively such as Vitamin D [6], bisphosphonates [7], selective estrogen receptor modulators [8] and RANK ligand inhibitors [9, 10]. Furthermore, therapeutic strategies targeting both are of considerable interest and has attracted much attention. Strontium ranelate has been developed to support the idea by concomitantly inducing osteoclasts apoptosis and osteogenic pathway activation through binding with calcium-sensing receptor [11, 12].

In recent years, inorganic nanomaterials have been intensively explored to prevent the bone degeneration [13, 14], especially with the natural inorganic components such as Ca, Mg, Zn, Fe, Si, Sr [15−23], exhibiting acceptable safety profiles and bioactive functions. Considerable studies have proved that the inorganic nanomaterials inhibit the bone loss mainly by facilitating the osteoblast differentiation and mineralization. Among them, hydroxyapatite nanoparticles (HANPs) are the most famous ones as their biomimetic components (Ca_5_(PO_4_)_3_(OH)) and nano size [24, 25]. Moreover, several cellular signaling pathways including wnt/β-catinin [26], TGF-β/Smad [27], DNA methylation [28], calcium homeostasis [29] have been verified involving in the process of HANPs promoted osteogenesis. Beyond osteogenesis promotion, the inorganic nanomaterials also displayed osteoclastogenesis inhibition property in recent studies [20, 30, 31]. Thus, it was a reasonable strategy to integrate the bilateral advantage into one inorganic nanosystem for dual targeting osteogenesis promotion and osteoclastogenesis inhibition. However, similar nanocomposite systems with the above-mentioned dual functions have not been reported before.

According to the published works, iron oxide nanoparticles involved in osteoporosis treatment was mainly due to the iron supplementary or magnetic property [32−34]. In our previous study, the clinically used superparamagnetic iron oxide nanoparticles (SPIO@HA) were able to target bone defects and suppress osteoclastogenesis through TRAF6−p62−CYLD signaling complex regulation [30]. Thus, a continuous work is conceived on improved bone remodeling by simultaneously increasing bone formation and diminishing bone resorption. Herein, in this study, SPIO@HA nanocomposites were prepared by coating HA on PAA-coated SPIO in a relatively simple method. The optimal ratio of SPIO to hydroxyapatite (HA) in one nanosystem was rigorously screened for both promoting mesenchymal stem cell (MSC) oriented osteoblast differentiation and inhibiting bone marrow monocyte (BMM) oriented osteoclast differentiation. An ovariectomized bone loss mouse model was employed to evaluate the therapeutic effect of the nanosystem. Moreover, the possible underlined mechanism was studied through molecular biology and high throughput analysis.

## Materials and methods

### Synthesis of SPIO nanoparticles

The synthesis of SPIO was all in N_2_ atmosphere. A 3 mL solution of 0.31 g FeCl_3_·6H_2_O and 0.16 g FeCl_2_·4H_2_O was added with 80 μL acid solution (37% HCl). Then the mixture was quickly added to the alkaline solution (3.7% NH_3_·H_2_O) under vigorous stirring at least 30 s. Next, a 6 mL solution of 0.41 g polyacrylic acid (PAA, 2.0 kDa) solution was added dropwise to the above prepared solution and kept stirring for 1 h. Finally, the mixture was centrifuged at 4000 rpm for 30 min and the supernatant was collected and dialyzed with deionized water for 3 days to obtain PAA coated SPIO.

### Synthesis of HA nanoparticles

A 30 mL solution of 0.05 g PAA was stirred while maintaining the temperature at 80°C and pH at 10. Then a 10 mL solution of 0.09 g Ca(NO_3_)_2_·4H_2_O was gently added to the PAA solution and keep the pH at 10 for 0.5 h. Next, a 30 mL solution of 0.034 g Na_2_HPO_4_ was added dropwise into the mixture and keep the pH at 10 for 8 h. The final HA suspension was obtained after 3 days dialysis in deionized water.

### Synthesis of HA-coated SPIO@HAs

For synthesis of SPIO@HA, 30 mL SPIO aqueous solution containing 1.4 mg Fe was stirred while maintaining the temperature at 80°C and pH at 10. Then the preparation of SPIO@15HA, SPIO@5HA and SPIO@3HA were using a 10 mL solution of 90 mg, 30 mg, 18 mg Ca(NO_3_)_2_·4H_2_O (at Fe/Ca mole ratio of 1:15, 1:5, 1:3), respectively. The Ca(NO_3_)_2_ solution was gently added to the SPIO solution and maintain the pH at 10 for 0.5 h. Next, Na_2_HPO_4_ solution with Ca/P = 1.67 was added into the mixture and maintain the pH for another 8 h. The final SPIO@HA suspension was obtained after 3 days dialysis with deionized water.

### Physicochemical characterization

Transmission electron microscopy (TEM) analysis was performed on an FEI Tecnai 20 microscope (FEI, US) at 100 kV. One drop of dispersed nanoparticles was added on the surface of a copper grid, dried at room temperature and then examined by TEM. Elemental analyses were performed by energy-dispersive spectrometer (EDS) on INCAPentaFETx3 (Oxford Instruments, UK). The size and zeta potential of all samples were tested by dynamic light scattering (DLS) on a Malvern Nanosizer (Zetasizer Nano ZS, UK). Fourier transform infrared (FTIR) spectra were delineated on a Perkin-Elmer spectrophotometer in the region of 4500 − 0 cm^−1^ with a powder sample on a KBr plate. X-ray diffraction (XRD) patterns were collected on a Dandong Fangyuan DX-1000 diffractometer (Haoyuan Instrument, China) with a Cu Kα radiation source (λ = 1.5418 Å) in the 2θ range 20°−80°. Iron and calcium concentration of all samples were evaluated by elemental analysis using an atomic absorption spectroscopy (AAS) (AA800, Perkin-Elmer, US). *T*_2_ relaxivities were recorded and calculated on a 1.5 T clinical magnetic resonance imaging (MRI) scanner (Siemens, Germany) at room temperature.

### Cell culture

For BMMs extraction, bone marrow cells were firstly flushed out from femur and tibia from female BALB/c mice and added into a 10 cm dish. The suspended cells were collected after 24 h culture, leaving behind the attached cells. Then the extracted cells were cultured in α-MEM medium (10% FBS) with 20 ng/mL macrophage colony-stimulating factor (M-CSF) (PeproTech, USA) for 48 h. The remaining cells, mainly pre-osteoclast cells, were added into culture plate with 50 ng/mL M-CSF and nuclear factor κB ligand (RANKL) (PeproTech) for further osteoclast differentiation at suitable cell densities. For bone MSCs culture, cells were firstly isolated from both femur and tibia of female SD rats and seeded into the 10 cm dish. After 48 h incubation, the suspended cells were washed away, leaving only the adherent cells (Passage 1). The MSCs were further passaged every other 2 days and Passage 3 were used for further osteoblast differentiation at appropriate cell densities.

### Cell viability and proliferation assay

Cell viability and proliferation were determined by colorimetric assay with cell counting kit (CCK-8, Dojindo, Japan). Briefly, BMMs and MSCs were treated with different concentrations of nanoparticles in 96-well plates. The concentrations were calculated by the amount of iron (Fe: μg/mL), and the HA concentrations of HA were equal to that of SPIO@HA nanocomposites. Ten percent of CCK-8 assay solution was added to the medium at the indicated times and incubated for a further 2 h. The optical density (OD) values of the wells were measured on a microplate reader at 450 nm.

### Intracellular iron content assay

The nanoparticle uptake ability of cells was determined by the colorimetric ferrozine assay refer to our previous work [35]. MSCs and BMMs (2 × 10^4^ cells/well) were cultured in a well of 48-well plate and treated with varied dosage of nanocomposites for 24 h. Then the cells were rinsed with PBS for three times and dissolved with NaOH solution (50 mM) for 2 h. After neutralized with HCl (10 mM), the cells were incubated with iron-releasing reagents at 60°C for another 2 h. Then iron detection reagent was added and reacted for 30 min. The ODs were measured on a microplate reader at 550 nm. The intracellular iron contents were calculated against the standard curve according to OD values.

### Alizarin red-S (ARS) staining assay

MSCs (5 × 10^3^ cells/well) were seeded into a 96-well plate and incubated with or without varied dosage nanocomposites. The medium was replaced every 3 days in the presence or absence of nanoparticles. After 7 days, the cells were fixed with 4% paraformaldehyde for 10 min and stained with 2% Alizarin red-S solution (Sigma, USA) which dissolved in deionized water for 20 min. Then the cells were observed under a light microscope. ARS quantification was determined by the percentage of ARS-positive area in total area measured by image J software.

### Tartrate-resistant acid phosphatase staining

BMMs (5 × 10^3^ cells/well) were seeded in 96-well plates and treated with M-CSF (50 ng/mL) for 24 h, RANKL (50 ng/mL) was then added to stimulate osteoclasts formation with or without nanoparticles incubation at indicated concentrations. The medium was replaced every 3 days in presence or absence of nanoparticles together with M-CSF and RANKL. After 4 days, cells were fixed by 4% paraformaldehyde for 15 min and stained with tartrate-resistant acid phosphatase (TRAP) detection kit (387A-1KT, Sigma). Then the cells were captured under a light microscope. TRAP-positive cells with more than five nuclei were counted as osteoclasts.

### Actin ring formation assay

BMMs (1 × 10^4^ cells/well) were seeded a well of 48-well plates and stimulated by M-CSF and RANKL with or without the nanoparticles incubation as previously described. After 4 days incubation, cells were fixed in 4% paraformaldehyde and permeabilized in 0.1% Triton X-100, washed in PBS and stained with rhodamine-conjugated phalloidin overnight at 4°C (P1951, Sigma). The actin rings were visualized and captured by a fluorescence microscope.

### Real-time PCR

BMMs were detached after 4 days coculture to extract total RNA with PureLink RNA Mini Kit (Thermo, USA). Sample concentration and purity were assessed using a NanoDrop UV spectrophotometer (Thermo). Then PrimeScript^TM^ RT reagent Kit (Takara, Japan) was used for reverse-transcription and TB Green Premix Ex Taq^TM^ II (Takara) for Real-time PCR carried out by PCR Cycler QuantStudio 3 (ABI, USA). The expression of GAPDH was chose to normalize the result. The primer sequences of related genes were listed as followed:

mNfatc1, 5’-ATGGGGTCCCTATCAAGT-3′ (sence)
5’AGAAGTGGGTGGAGTGGT-3′ (antisense)
mSrc, 5′-TCTATCCCAGACACGACC-3′ (sense)
5′-AAACCAGACAGTTGAGGC-3′ (antisense)
mCalcr, 5′-GTAAGTGCCATTAGAGCG-3′ (sense)
5′-GGTAGGAGCCTGAAGAAC-3′ (antisense)
mRANK, 5’-ATCATCTTCGGCGTTTAC-3′ (sense)
5′-CTTCTTGCTGACTGGAGG-3′ (antisense)

### Western blot

BMMs were stimulated with 100 ng/mL RANKL at different time points after coculture with SPIO@15HA for 24 h, then lysed by RIPA lysis buffer (Cell Signaling Technology, US) added with protease inhibitors cocktail (Roche, Switzerland). Cellular proteins were dissolved and centrifuged at 12 000 rpm for 15 min at 4°C to get the supernatants. The concentrations of proteins were examined using Micro BCA^TM^ kit (Thermo). Equal amount of proteins from different group was loaded onto a SDS-PAGE electrophoresis gel (80 V, 2 h) and transferred to a polyvinylidene fluoride membrane (100 V, 40 min). The membrane was then blocked in PBS with 5% non-fat milk and incubated with primary antibodies against p62 (ab56416, Abcam, UK) and β-actin (sc-47778, Santa Cruz Biotechnology, US) overnight at 4°C. After thoroughly washing with PBST (0.1% Tween-20 in PBS), the membrane was incubated with HRP conjugated secondary antibody (#7076, Cell Signaling Technology, US) for 1 h at room temperature. The protein band was color reacted by BeyoECL Plus kit (Beyotime, China) and captured with ChemiDoc^TM^ XRS + System (Bio-Rad, USA).

### Gene expression profile microarray

MSCs were incubated with or without SPIO@15HA (Fe: 100 μg/mL) for 3 days. The cells were then washed with TRIzol reagent (Thermo) for total RNA extracting and microarray analysis was done by KangChen Bio-tech. Briefly, the RNA of each sample was labeled with Quick Amp Labeling Kit (Agilent, USA) and hybridized with a hybridization oven (SureHyb, Agilent). The array signals were scanned using the DNA Microarray Scanner (G2505C, Agilent), coupled with GeneSpring GX v12.1 software (Agilent).

### *In vivo* experiments

All animal procedures were conducted under the approvement of the Biomedical Research Ethics Committee of West China Hospital of Sichuan University. Female C57BL/6 mice (6 − 8 weeks old) were purchased from Chengdu Dashuo Experimental Animals co. LTD. For pharmacokinetics evaluation of nanoparticles, 12 mice were divided into 3 groups with intravenous injection of saline, SPIO@15HA (10 mg Fe/kg) and SPIO (10 mg Fe/kg), respectively. Blood and organs of mice were harvested following euthanasia after the injection for 4 days. Supernatant serum was taken after the whole blood coagulated overnight followed with centrifugation at 5000 rpm for 10 min at 4°C. Then liver function (alanine aminotransferase (ALT), aspartate transaminase (AST), alkalinephosphatase (ALP), albumin (ALB), total bilirubin (TBIL) and total protein (TP)) and kidney function (cholesterol (CHO), creatinine (CREA), urea (UREA) and glucose (GLU)) examinations were carried out by automatic biochemical analyzer Chemray 240 and 800 (Rayto, China). Heart, liver, spleen, lung, kidney and bone were fixed in 4% paraformaldehyde for 2 days and subjected to H&E staining for biosafety evaluation. Osteoporosis model was created by the bilateral ovaries excision of mice and sham group were executed all the operative procedures without ovary excision. After 1 week-recovery and antibiotic therapy, 14 ovariectomized (OVX) mice were divided into two groups with SPIO@15HA (10 mg/kg) and saline intravenous injection every 4 days. Sham group (7 mice) was also treated with saline every 4 days. The mice weight was recorded weekly throughout the whole study. After 90 days treatment, serum was obtained for biochemical examinations and key organs were fixed as described above. To assess trabecular bone areas, femurs were collected and fixed in 4% paraformaldehyde for at least 1 week. The Micro-computed tomography (micro-CT) study of fixed femurs was performed by Scanco vivaCT80 scanner (Scanco Medical, Switzerland) at 55 kVp and 145 μA with voxel size of 8 μm. The bone mass was calculated and evaluated by Scanco analytical software to obtain trabecular density and architectural parameters, together with 3D reconstructed images.

### MR Imaging examination

Two female Sprague-Dawley (SD) rat (7-week-old) were injected with SPIO@15HA and SPIO (Fe, 10 mg/kg) in caudal vein, respectively, for MRI. The study was performed by clinical 3 T Skyra scanner (Siemens) with a rat coil using *T*_2_-weighted spin echo sequence *tse2d1rr6 (TR = 2500 ms, TE = 68 ms; matrix = 192 × 192, field of vision = 70 × 70 mm, slice thickness = 1.0 mm, flip angle = 150°). The rats were anesthetized by inhalation of isoflurane (3 − 4%) delivered through respiratory masks during the whole imaging procedure. *T*_2_-map images was calculated by different MRI intensity at a series of TE values of each phantom (TE = 11 − 68 ms).

### Immunohistochemical staining

The fixed heart, liver, spleen, lung and kidney of experimental animals were dehydrated in gradient alcohol, embedded by paraffin wax and cut into 4 μm sections. Femurs were firstly decalcified in 10% ethylenediaminetetraacetic acid for 3 weeks before subjecting to the operations of preceding step. Following that, H&E and TRAP staining as well as osteoprotegerin (OPG) and bone morphogenetic protein 2 (BMP2) immunological staining were performed on tissue sections. Briefly, sections were firstly deparaffinized by xylene and ethanol. After that, H&E and TRAP staining were performed with the reagent instructions. For OPG and BMP2 immunohistochemical (IHC) staining, sections mainly went through procedures of endogenous peroxidase activity blocking, antigen retrieval, normal goat serum blocking, primary antibody incubation, HPR-conjugated secondary antibody incubation, 3,3-diaminobenzidine tetrahydrochloride reaction (Beyotime) and hematoxylin counterstaining. The IHC positive signals were measured by plugin IHC Profiler on ImageJ software.

### Statistical analysis

Statistical data were presented as means ± SD. Two-way analysis of variance (ANOVA) was used in experiments with groups at multiple time points and one-way ANOVA was used in the rest of experiments. All statistical analysis was performed with GraphPad Prism software. A *P* values <0.05 was considered statistically significant (*P < 0.05, **P < 0.01, ***P < 0.001).

## Results and discussion

### SPIO@HA core/shell nanocomposite synthesis and characterization

**Figure 1.**
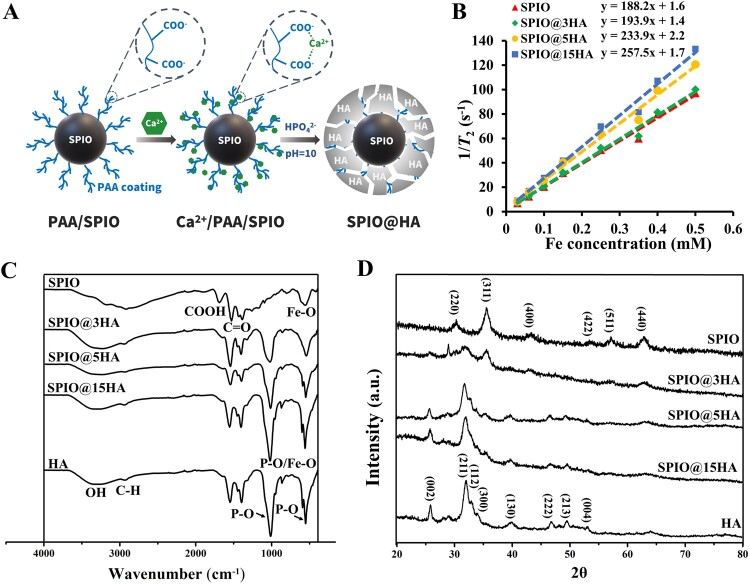
Synthesis and characterization of hydroxyapatite coated superparamagnetic nanoparticles (SPIO@HA). (**A**) Schematic illustration of SPIO@HA fabrication. (**B**) *T*_2_ relaxation rate (1/*T*_2_, s^−1^) as a function of Fe concentration (mM) for different SPIO@HA and SPIO nanoparticles under a 1.5 T magnetic field. (**C**) FTIR spectra and (D) XRD analysis of HA, SPIO and SPIO@HA at indicated ratios

According to our and others’ studies, the iron oxide core has a critical role for SPIO induced macrophage phagocytosis [36], autophagy response, immune reaction [37] and osteoclastogenesis inhibition [30]. Thus, the exposure of the bioactive core is important during the HA incorporation for osteogenesis promotion. A polymer material, PAA, was employed for high content but stable HA incorporation by its rich carboxyl groups [38], facilitating a loose surface formation for core exposure. PAA coated SPIO was synthesized by the coprecipitation method followed with Ca(NO_3_)_2_ and Na_2_HPO_4_ addition at molar ratio of 1.67:1 to obtain SPIO@HA (Fig. 1A). SPIO@HA with different content of HA coatings were acquired by adjusting the amount of the added Ca(NO_3_)_2_ and Na_2_HPO_4_. In FTIR analysis, both the characteristic peaks of HA and SPIO appeared in three SPIO@HA composites with different shell thickness. Peaks at 3450 cm^−1^ were attributed to the OH and at 1048 and 570 cm^−1^ were attributed to the P−O of HA, while peaks at 570 cm^−1^ were attributed to the Fe−O of SPIO (Fig. 1C). In addition, the typical characteristic peak of SPIO (near 30° (220), 35.5° (311)) and HA ((002), (211), (112), (300)) crystalline structure were detected in the XRD spectrum of obtained SPIO@HA nanocomposites (Fig. 1D). The peak intensity of HA and SPIO showed content-dependent increase in SPIO@HA. Next, the *r*_2_ relaxivity of all samples were measured under clinical MRI scanner at 1.5 T (Fig. 1SA), showing a HA content dependent increase from 188.2 to 257.5 mM^−1^s^−1^ (Fig. 1B). The HA-coating with loose structure may cause the diffusion of water molecules restricted in the HA framework, resulted in the effective electronic spin relaxation of water protons, which is similar to the results of previously reported inorganic materials (such as mesoporous silica and titania)-coated SPIO [39, 40].

**Figure 2.**
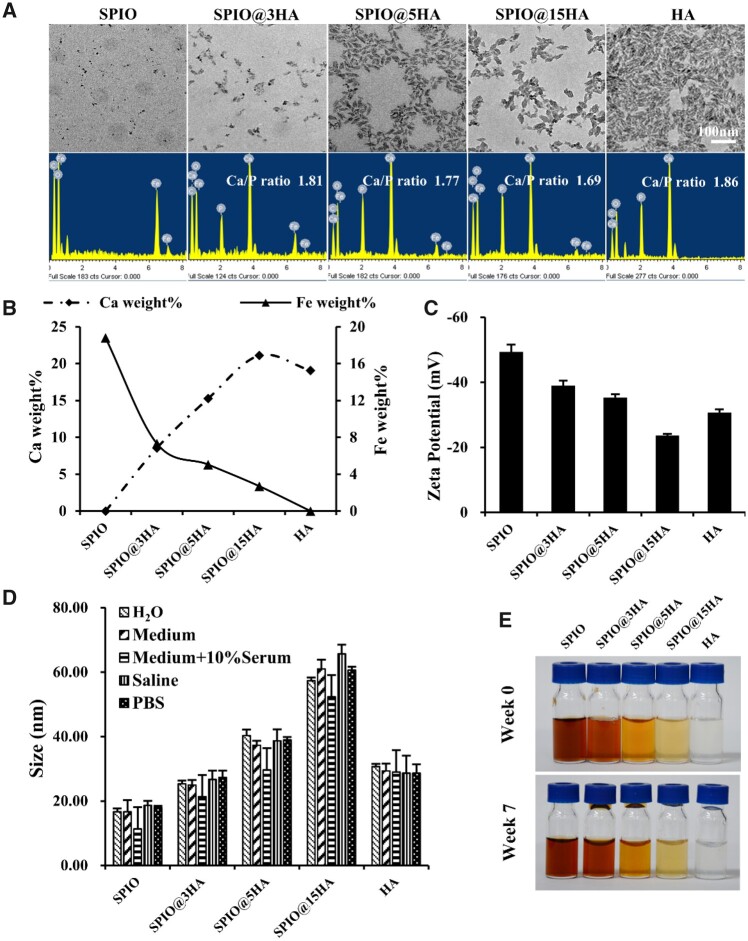
Physicochemical characterization of SPIO@HA. (**A**) TEM images and EDS analysis of as-synthesized nanoparticles. (**B**) Ca and Fe mass percentage of total mass in SPIO@HA nanocomposites calculated from EDS results. (**C**) Zeta potential of SPIO@HA by DLS analysis. (**D**) Average particle size of nanoparticles in different medium by DLS analysis. (**E**) Photos of SPIO@HA nanocomposites stability in deionized water for 7 weeks, room temperature. HA, SPIO@3HA, SPIO@5HA and SPIO@15HA at hydroxyapatite concentration of 20 mg/mL, SPIO at iron concentration of 0.5 mg/mL

In accordance to TEM results, the HA and SPIO@HA composites have spindle-like morphology and narrow size distribution. The SPIO@HA composites showed an iron oxide core with spherical shape. The EDS spectrum data further verified a successful HA preparation with Ca/P ratio around 1.67 (Fig. 2A). The particle size of SPIO@HA increased while its zeta potential decreased with gradually increased HA content (Fig. 2B−D and Supplementary Fig. S6). Introduction of HA shell led to a larger particle size, which was proportional to the added amount of calcium and phosphate salt. At the same time, the shell shielded partial negative charge of iron oxide core, changed from −49 to −24 mV when SPIO compared with SPIO@15HA. The nanoparticles in all samples displayed good stability and dispersibility in different solvents including deionized water, cell culture medium, cell culture medium with 10% serum, saline and PBS (Fig. 2D, E and Supplementary Fig. S1B). Among them, particle size in the cell culture medium with 10% serum group exhibited a decline trend may contribute to the serum protein disturbance.

### SPIO@HA inhibited bone BMM oriented osteoclast differentiation and promotes MSC oriented osteoblast differentiation

**Figure 3.**
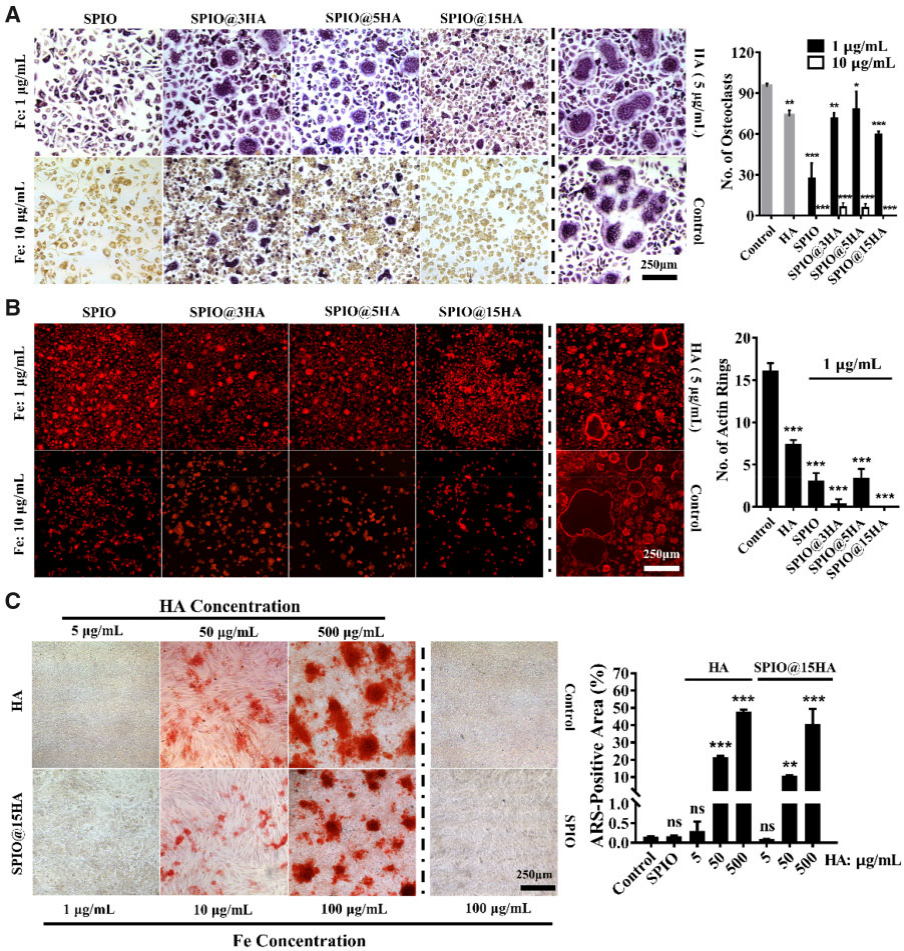
SPIO@HA Inhibited BMM oriented osteoclast differentiation and promotes MSC oriented osteoblast differentiation. (**A**) BMMs were incubated with the indicated concentrations of as-synthesized nanoparticles for 4 days under the stimulation of RANKL and M-CSF. TRAP staining was carried out and the number of osteoclasts was counted. (**B**) BMMs were incubated with the indicated concentrations of as synthesized nanoparticles for 4 days under the stimulation of RANKL and M-CSF. Rhodamine-conjugated phalloidin staining was carried out and the formation of actin ring was quantified. (**C**) MSCs were incubated with the indicated concentrations of HA, SPIO@15HA and SPIO nanoparticles for 7 days, then alizarin red-S staining was carried out and the positive area was quantified. Cells without nanomaterials addition was served as the control group. Values are expressed as the mean, *n* = 3; bars show SD; significant analysis was performed vs control group; ns means no significance. * *P* < 0.05, **P < 0.01, *** *P* < 0.001

TRAP staining, the hallmark method of studying osteoclast differentiation was employed to detect effect of SPIO@HA on BMM oriented osteoclasts. As shown in Fig. 3, PAA encapsulated SPIO obviously suppressed the differentiation even at a very low iron dosage of 1 μg/mL, more efficient than previously reported dextran coated SPIO at 10 μg/mL [30]. Meanwhile, the HA also showed slightly inhibitory effect on the differentiation. However, the HA incorporation lowered the inhibitory effect of SPIO in nanocomposites of SPIO@3HA, SPIO@5HA and SPIO@15HA. Among them, SPIO@15HA exhibited the most obvious inhibitory effect with the lowest internalization (Supplementary Fig. S2D). Actin ring is an essential structure for bone resorption. The sealing zone formed by the rearrangement of actin in active osteoclasts appeared as a red circle under a fluorescence microscope and was counted as actin ring with the diameter >20 μm. Same results were obtained in the actin ring staining assay, representing the suppression of osteoclasts resorption activity by nanoparticles (Fig. 3B). After that, bone marrow MSCs were extracted and treated with SPIO@15HA at different HA concentration (Fig. 3C). PAA encapsulated HA nanoparticles could successfully induce MSC osteoblast differentiation in a dosage dependent mode with HA concentration of at least 50 μg/mL after 7 days’ treatment. While at the same amount of SPIO@15HA, Fe concentration was 10 μg/mL, which can totally suppress the BMM osteoclast differentiation. As a control group, SPIO itself had no effect on the differentiation, indicating that SPIO@15HA induced MSC osteogenesis was HA content dependent. The results supported the conclusion that SPIO@15HA have dual function on inhibiting osteoclastogenesis mainly by SPIO while promoting osteogenesis mainly by HA. Besides, no obvious cytotoxicity of SPIO, HA and SPIO@HA was found in both BMMs and MSCs groups (Supplementary Fig. S2).

### MRI *in vivo* study of SPIO@15HA targeting to the rat bones

**Figure 4.**
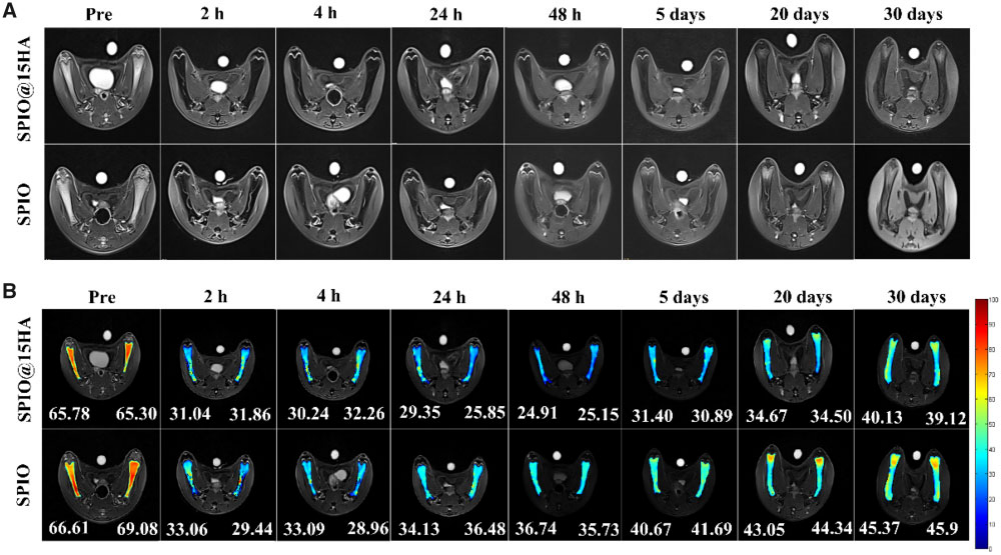
Accumulation of SPIO@15HA and SPIO nanoparticles in femoral bone marrow. (**A**) *T*_2_-weighted MRI images of rat femurs before or after SPIO@15HA and SPIO injection through tail vein at indicated time points. (**B**) *T*_2_-map images of rat femurs calculated by different MRI intensity from *T*_2_-weighted MRI images

With the evident function of inhibiting osteoclastogenesis as well as promoting osteogenesis, SPIO@15HA was then selected for *in vivo* study. As a member of endothelial reticular system, bone resident macrophages could efficiently trap injected nanomaterials [41], facilitating their targeting route to bone defects. MRI was employed for SPIO@15HA bone targeting visualization on account of good relaxivity of nanoparticles. *T*_2_-weighted images of rat femur showed that SPIO@15HA had faster and sharper drops on *T*_2_ signal intensity than SPIO after intravenous injection (Fig. 4A). *T*_2_-map imaging also showed that the minimum average *T*_2_ value dropped to 25.03 ms at 48 h in SPIO@15HA injected mouse followed with gradual signal intensity value recovery. Even after 30 days, an average *T*_2_ value of 39.63 ms was still observed in SPIO@15HA injected mice, much lower than SPIO treated one (45.67 ms). The results indicated that SPIO@15HA have more accumulation than SPIO probably due to the HA shell coating prolonged SPIO degradation.

**Figure 5.**
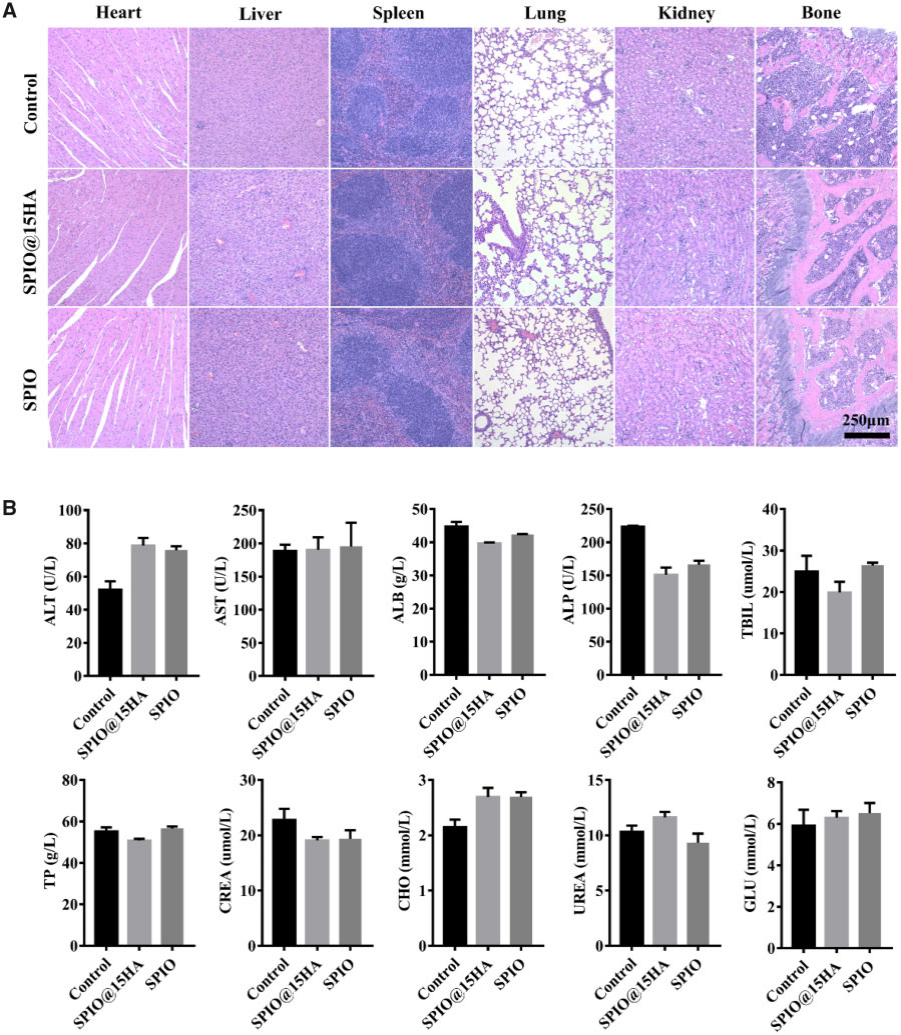
Biosafety study of SPIO@15HA and SPIO via tail vein injection. (**A**) H&E staining of main organs from mice 4 days after treatment with saline (control), SPIO@15HA and SPIO. (**B**) Serum levels of liver and renal function related indexes of mice 4 days after treatment with saline (control), SPIO@15HA and SPIO

To further evaluate the biosafety of injected nanomaterials, serum and major organs of mice were harvested and subjected to histologic assessment after exposure to SPIO@15HA and SPIO. No obvious histopathological changes were observed in H&E staining of tissue sections (Fig. 5A). Serum indexes of liver and kidney function assay did not show any significant change compared with the untreated group (Fig. 5B).

### SPIO@15HA prevented bone loss in ovariectomized (OVX) mice

**Figure 6.**
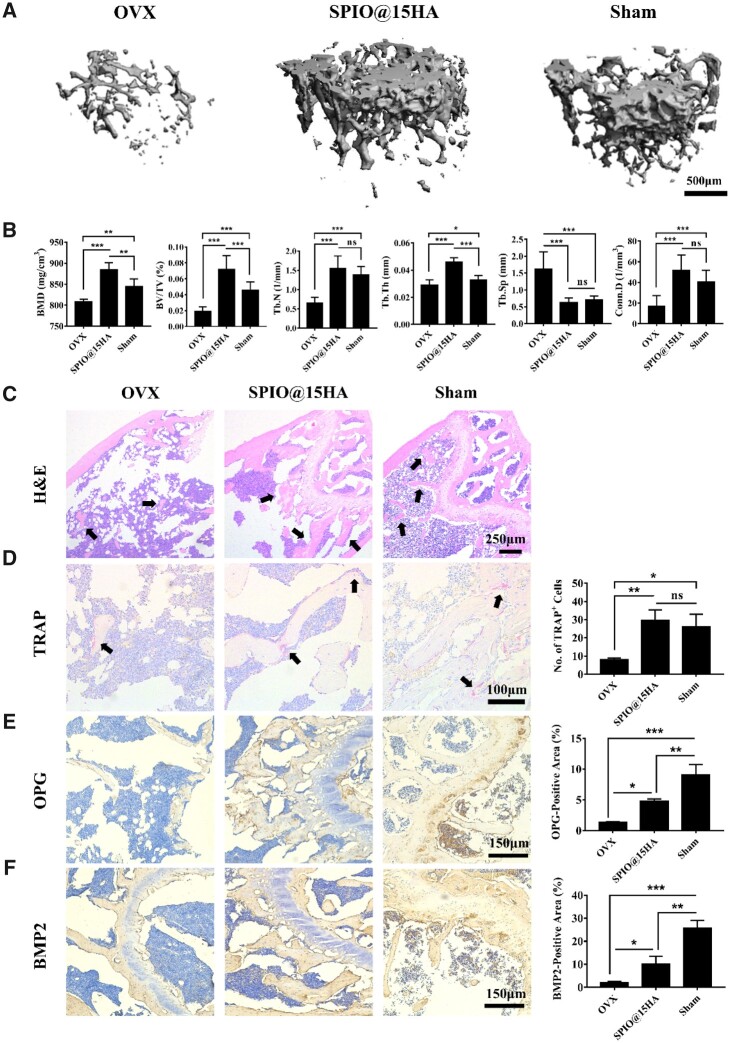
SPIO@15HA Prevented OVX-induced bone loss. (**A**) 3D micro-CT reconstruction images of trabecular bone in each group, representative of sham, OVX and OVX treated with SPIO@15HA. (**B**) Trabecular bone mass parameters. *n* = 7 (seven mice in each group). (**C**) H&E staining of femoral sections in each group, showing reduced trabecular bone (black arrow). (**D**) TRAP staining showing decreased TRAP activity in OVX mice (black arrows) and counting of TRAP positive (TRAP^+^) cells. (**E**) Immunohistochemical staining against OPG and quantification of positive area. (**F**) Immunohistochemical staining against BMP2 and quantification of positive area. *n* = 3 (three mice in each group). Values are expressed as the mean; bars show SD. ns means no significance. *P < 0.05, **P < 0.01, *** *P* < 0.001

Inspired by efficient bone targeting and reasonable *in vivo* safety profile of SPIO@15HA, an ovariectomized (OVX) mouse model of osteoporosis was then developed to evaluate the therapeutic effect of SPIO@15HA on bone loss. Ovariectomy usually causes a sharp decline of estrogen levels in mice, leading an imbalance between the activity of osteoblasts and osteoclasts, is a classic animal model to mimic osteoporosis in postmenopausal women. After three months’ treatment, femur bones were excised from mice and fixed with formalin and then subjected to micro-CT scan for assessment of bone mass, morphology and microarchitecture. As shown in 3 dimension (3D) reconstructed images with micro-CT, the images of caput femoris showed severe bone loss in OVX mice after surgery at 3 months, while the treated and sham groups exhibited much denser femoral metaphyseal structure (Fig. 6A). The bone morphometric parameters of 3D construction gave more quantitative demonstration. The bone mineral density (BMD) and bone volume fraction (BV/TV) in SPIO@15HA group was significantly increased even more than a normal value showed in the sham group. The key trabecular bone parameters, trabecular number (Tb. N), trabecular thickness (Tb. Th) and connectivity density (Conn. D) of SPIO@15HA group were all displayed the same trend. Trabecular separation (Tb. Sp) in SPIO@15HA treated mice were significantly lower than OVX treated one (Fig. 6B). The body weight of OVX mice showed a slight increase during the treatment (Supplementary Fig. S3). This might be a sign of early osteoporosis, for the mice were less physically active because of bone loss, resulting weight gain. The H&E staining of femurs in each group corroborated the micro-CT results (Fig. 6C). The OVX group showed sever trabecular bone loss compared with the sham group as well as the SPIO@15HA treated one, which displayed relatively intact trabecular structure. The results further indicated treatment with SPIO@HA could prevent ovariectomy-induced osteoporosis. Interestingly, femoral section of SPIO@15HA and sham group contained more TRAP-positive osteoclasts than the OVX group (Fig. 6D), that probably attributed to the huge trabecular bone loss in the OVX group, leading to the lack of cell adhesion surface for osteoclasts. In the meantime, both osteoblasts and osteoclasts showed strong activity to maintain bone metabolism balance in SPIO@15HA and sham group. Immunohistological staining of OPG and BMP2 were performed and quantified as they played important roles in bone remodeling activities (Fig. 6E and F). The SPIO@15HA treated ones partially recovered the expression relevant to the sham group. Although SPIO@15HA demonstrated a great potential in preventing bone loss of the OVX mice according to micro-CT analysis and femur histology staining results, but it presented some limitations in terms of therapeutic doses given to mice. Large accumulation of nanoparticles in liver (Supplementary Fig. S3A) resulted in abnormal increases of ALT and AST after 3 months (Supplementary Fig. S3B), indicating impaired liver function of mice with a long-term SPIO@15HA injection. However, obvious tissue impairment was not observed in tissue section H&E staining (Supplementary Fig. S3A). Further work is required to identify a better therapy paradigm of SPIO@15HA in osteoporosis treatment.

### SPIO@15HA nanocomposites up-regulated p62 expression in preosteoclasts and activated osteogenesis associated signaling pathways in MSC

**Figure 7.**
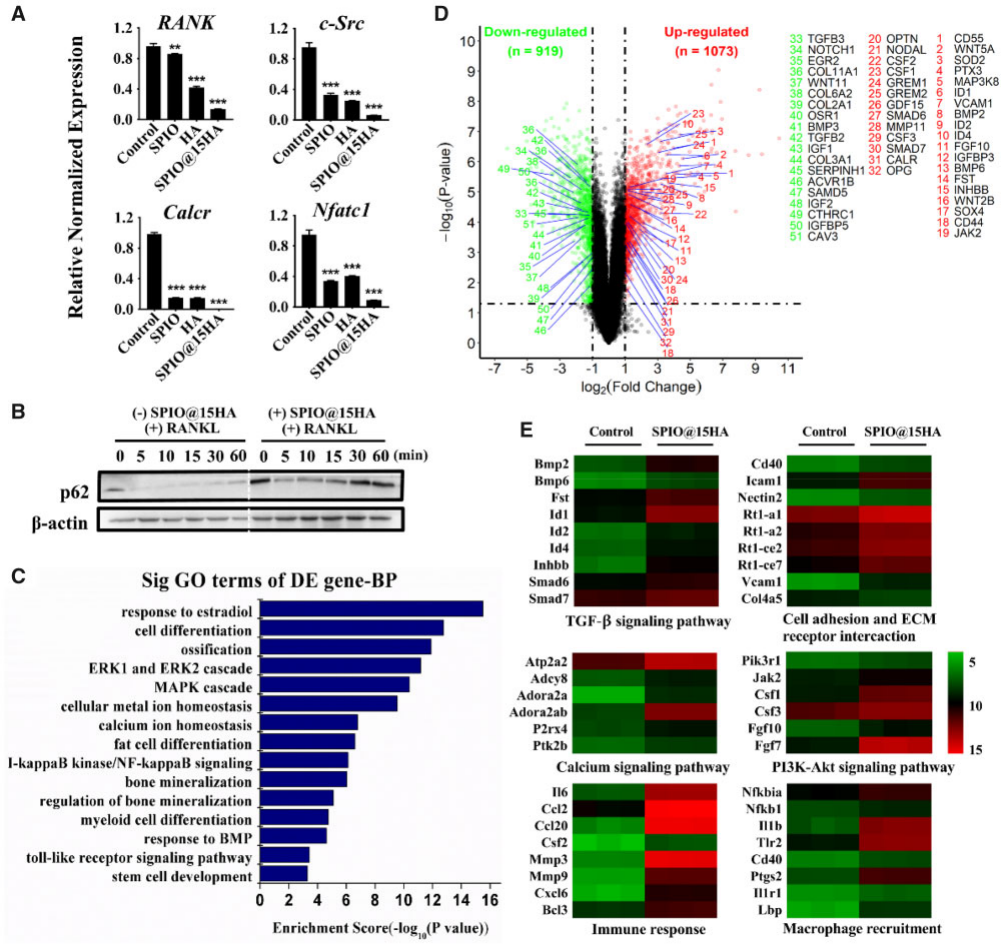
Signaling pathways related to osteoclastogenesis and osteogenesis were regulated by SPIO@15HA nanocomposites. (**A**) Expression levels of osteogenesis-related maker genes in nanoparticles treated osteoclasts at the fourth day of differentiation stage. Osteoclasts without nanoparticle treatment were served as the control group. Fe concentration of nanoparticles were 1 μg/mL. Values are expressed as the mean; bars show SD. (**B**) Western blotting analysis of p62 expression in osteoclasts with RANKL stimulation at indicated time points and cells were pretreatment with SPIO@15HA at Fe concentration of 1 μg/mL for 24 h. (**C**) Volcano plot showing differentially regulated genes in SPIO@15HA treated MSCs when compared with the nontreated one. Down- and up-regulated genes closely related to MSC osteogenesis with a fold change of >2 at *P* values <0.05 are highlighted in green and red, respectively. (**D**) Gene ontology enrichment analysis of differentially expressed coding genes in BP connected with osteogenic differentiation (−log10 (*P* values) > 3). (**E**) Heat map showing genes involved in osteogenic pathways that were expressed differently in SPIO@15HA treated MSCs when compared with the nontreated control. The fold-change ratio is greater than or equal to 2 and was calculated, *P* < 0.05. Green and red colors represent the relative fold change of downregulated and upregulated gene expression, respectively. *n* = 3

During the process of osteoclast maturation, several marker genes play the pivotal roles including: RANK, the initial signal of osteoclast formation [42, 43]; c-Src, essential in osteoclast polarizing and ruffled-membrane formation [44]; Calcr, involved in the bone resorption [45]; and NFATc1, the crucial regulator of osteoclast differentiation in multiple phases [46]. RT-qRCR was carried out to explore whether the nanoparticles have effect on expression of these genes. SPIO, HA and SPIO@15HA could notably suppress marker genes expression during osteoclastogenesis stimulated by RANKL and M-CSF (Fig. 7A), showing the capability to inhibit osteoclast differentiation.

**Scheme 1.**
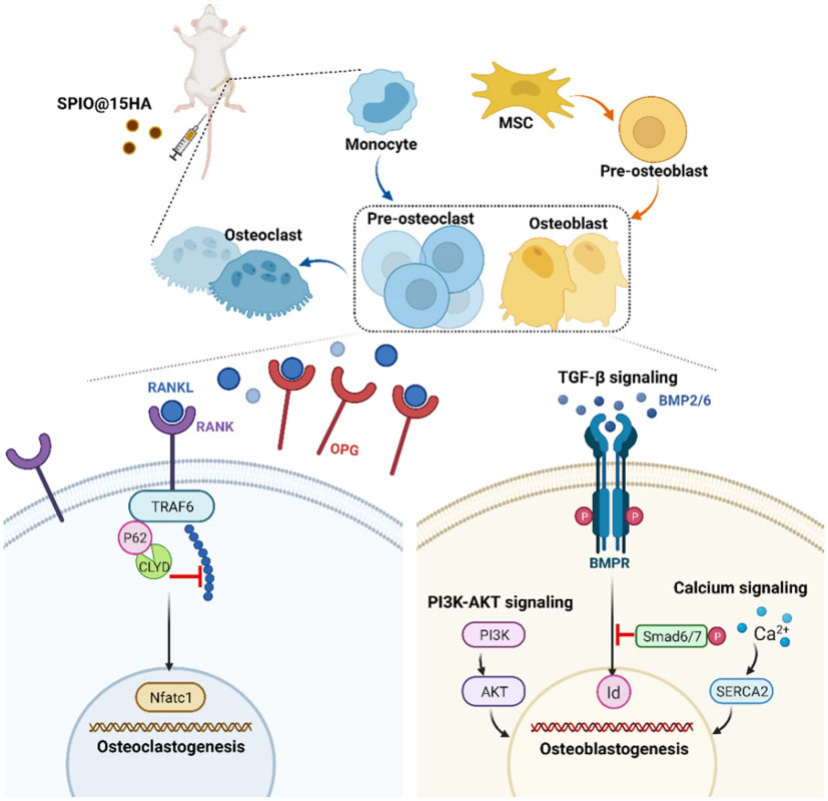
Illustration of SPIO@15HA nanocomposites’ impacts on BMMs oriented osteoclastogenesis and MSCs oriented osteogenesis. After tail vein injection, SPIO@15HA nanocomposites were accumulated in bone marrow and affected the bone remodeling process by suppressing osteoclastogenesis and stimulating osteogenesis. SPIO@15HA blocks RANKL induced osteoclastogenesis of BMMs through p62 upregulation resulted deubiquitination of TRAF6 by over CYLD recruitment, thus leading to the signal transduction termination of RANKL activated pathway. SPIO@15HA also increases the expression of OPG by MSCs, which competitively inhibits RANKL/RANK combination. On the other side, TGF-β, PI3K-AKT signaling and calcium homeostasis related to osteogenic differentiation are activated in MSCs when interacting with SPIO@15HA nanocomposites

In our previous work, we have found that clinically used SPIOs can increase p62 expression though TLR4 activation [37], thus induce recruitment of CYLD to inhibited TRAF6 ubiquitination, leading to suppression of RANKL induced signal transduction for osteoclast differentiation [30]. We then examined p62 expression with or without SPIO@15HA incubation. The results showed that p62 expression was obviously upregulated after 24 h nanoparticle incubation (Fig. 7B). Moreover, RANKL induced p62−CYLD−TRAF6 complexes ubiquitination degradation was inhibited, which may also cause the downstream signal transduction blockade (Fig. 7B).

To explore how SPIO@15HA nanoparticles involved in the osteogenic process of MSCs, a gene expression profile microarray was performed. Around 2000 genes were regulated in MSCs when treated with SPIO@15HA nanocomposites (*P* < 0.05, fold change > 2) compared with untreated one. In bone development related gene expression files, 186 up-regulated and 92 down-regulated genes were statistically analyzed by the heat map (Supplementary Fig. S5), indicating an evident osteogenesis regulation trend. Among the up-regulated and down-regulated genes related to bone development, the most important ones were labeled in the volcano plot (Fig. 7C), including BMP2, BMP6 [47], OPG [43], showing a similar trend in IHC assay as above described (Fig. 6E and F).

To investigate the correlation among signal pathways, GO analysis based on Gene Ontology and pathway analysis based on Kyoto Encyclopedia of Genes and Genomes (KEGG) was carried out. According to the enrichment scores of terms included in GO, multiple biological processes (BPs) connected with osteoblast differentiation were significantly up-regulated and top 15 of them were listed and most of them have been reported closely correlated with osteogenesis (Fig. 7D). Meanwhile, 73 up-regulated and 49 down-regulated pathways were found on basis of KEGG, of which the BMP/Smad signaling in TGF-β signaling pathway was well studied to enhance osteoblast differentiation [27, 48]. The other differentiated pathways related to MSCs osteogenesis predominantly could be categorized into four major components: (i) bone-specific differentiation: PI3K-Akt signaling pathway [49] and JAK-STAT signaling pathway [50]; (ii) cell migration and proliferation: cell adhesion molecules and ECM-receptor interaction; (iii) calcium ion homeostasis: calcium signaling pathway; (iv) immune response and macrophage recruitment: TNF signaling pathway, IL-17 signaling pathway, NF-kappa B signaling pathway and Toll-like receptor signaling pathway (Fig. 7E). These results suggested that SPIO@15HA nanoparticles may stimulate the osteoblast differentiation through TGF-β signaling pathway and calcium homeostasis. At the same time, SPIO@15HA may affect osteoblast−osteoclast interaction through increased macrophage recruitment by cytokine upregulation such as CSF-2 [51], IL-6 [52], CCL-2 [53], which deserved a further investigation to improve the therapy effect (Fig. 7C and E).

## Conclusions

In this study, a class of HA coated SPIO nanoparticles (SPIO@HA) with core−shell structure were successfully synthesized at varied Fe/Ca molar ratios, displaying good stability and low cytotoxicity in the *in vitro* studies. The nanocomposites at Fe/Ca molar ratio of 1:15 exhibited the dual regulated function on both osteoclastogenesis and osteogenesis. SPIO@15HA showed inhibitory effect on formation of RANKL-induced TRAP-positive multinucleate cells and actin rings during osteoclastogenesis. Meanwhile, it promoted MSC osteogenesis with the Alizarin red-S staining evaluation. MRI results demonstrated a longer bone marrow retention time of SPIO@15HA than SPIO, which may favor a better therapeutic effect. Prevention of osteoporotic bone loss *in vivo* was further validated after intravenous injection of SPIO@15HA to ovariectomized mice. Enhancement of bone trabecula microstructure was observed in SPIO@15HA treated mice by micro-CT analysis and tissue H&E staining. p62 up-regulation was demonstrated involving the osteoclastogenesis suppression. Meanwhile, TGF-β, PI3K-Akt pathways together with calcium homeostasis and cell motility signaling were analyzed, all of them serving significant roles for osteogenesis promotion. Besides, increased cytokine upregulation related to macrophage recruitment was also observed in SPIO@15HA treated MSC, indicating SPIO@15HA may have effect on osteoblast−osteoclast interaction which requires a further study. All the results indicated that SPIO@15HA nanocomposite was a good candidate for bone remodeling of osteoporosis by targeting both osteoclastogenesis and osteogenesis processes (Scheme 1).

## Supplementary data

Supplementary data are available at *REGBIO* online.

## Supplementary Material

rbab027_Supplementary_Data
