# Supplementary material for: Dual regulation of osteoclastogenesis and osteogenesis for osteoporosis therapy by iron oxide hydroxyapatite core/shell nanocomposites
Source: Regen Biomater. 2021 Aug 23;8(5):rbab027. doi: 10.1093/rb/rbab027 (PMC8382288; doi:10.1093/rb/rbab027)
Supplement: rbab027_Supplementary_Data [file rbab027_supplementary_data.docx]

Hua Ai, PhD, Professor	*Phone: 86-28-8541-3991*

National Engineering Research Center for Biomaterials	*Fax: 86-28-8541-3991*

Sichuan University, Email：huaai@scu.edu.cn

29 Wangjiang Rd., Chengdu, Sichuan 610064,

P. R. China

April 12, 2021

Dear Prof. Peppas,

We have carefully studied the comments from the reviewers for our work **"Dual Regulation of Osteoclastogenesis and Osteogenesis for Osteoporosis Therapy by Iron Oxide Hydroxyapatite Core/Shell Nanocomposites"** **(ID, RB-2021-033)**. Based on their comments and suggestions, we addressed all the questions and made corresponding changes in the revised manuscript.

***# Reviewer 1:***

In this manuscript entitled as "Dual Regulation of Osteoclastogenesis and Osteogenesis for Osteoporosis Therapy by Iron Oxide Hydroxyapatite Core/Shell Nanocomposites", authors have presented the development of hydroxyapatite coated superparamagnetic iron oxide nanoparticles (SPIO@HA) with a core-shell structure for targeting both osteoclastogenesis and osteogenesis. Overall this is an important study. This well-written manuscript would be very interesting to the readership of the Regenerative Biomaterials. I suggest the manuscript should be accepted. BTW, more explanatory statements can be added in the In vivo experiments to make it easier for understanding the ovariectomized (OVX) mouse model.

**Response:** We appreciate the reviewer’s comments and had added some explanatory of the ovariectomized (OVX) mouse model in the text and highlighted in yellow as the following: “Ovariectomy usually causes a sharp decline of estrogen levels in mice, leading an imbalance between the activity of osteoblasts and osteoclasts, is a classic animal model to mimic osteoporosis in postmenopausal women.”

***# Reviewer 2:***

Major comments:

1. Before studying the effect of SPIO@HA on the bone BMM and MSC oriented differentiation, it would be more reasonable to first discuss the biocompatibility of SPIO@HA to verify the safe concentration rage. In addition, in the viability experiment, it was unclear how the control groups were designed and whether the original culture medium containing SPIO@HA was removed before adding the CCK8 solution. From the photo appearance, the SPIO@HA at different Fe/Ca mole ratio had different absorbance property. It was not known whether the SPIO@HA had different absorbance property at the detecting wavelength or whether the SPIO@HA interfered the CCK8 reagent directly. Therefore, it would be better to provide more details about the method of the viability experiment.

**Response:** We appreciate the reviewer’s comments and made corresponding changes. Before studying the effect of SPIO@HA on the bone BMM and MSC oriented differentiation, we did have discussed the biocompatibility of SPIO@HA and the results were shown in the Supplementary Information (Fig. S2). The result showed that even at the highest iron concentration of 100 μg/mL, all the as-synthesized nanoparticles exhibited no significant cytotoxicity on MSC (Fig. S2B) and BMM after 24 h treatment (Fig. S2E). However, in evaluation of long-period cell growth, SPIO@3HA, HA and SPIO but not SPIO@15HA and SPIO@5HA exhibited proliferation inhibition compared to the untreated group at the iron concentration of 100 μg/mL (Fig. S2C). That is, all the concentrations of SPIO@15HA used in the following experiments of BMM and MSC oriented differentiation were in the safe range. In the viability experiment, the original culture medium containing nanoparticles would be firstly taken out and the cells would be then washed three times with PBS to remove the influence of SPIO@HA before adding the CCK8 solution. It needs to be explained that the iron concentration of different nanoparticles in the photos (Figure 2E and S1B) was set gradient on purpose to get a clear distinction of nanoparticles with different Fe/Ca mole ratio, which makes different absorbance of them in appearance. To eliminate the misunderstanding, we added the corresponding concentration of each nanoparticle in the description of Fig. 2E and Fig. S1B in the revised manuscript and highlighted them in yellow.

2. In Figure 3, the authors said that PAA encapsulated SPIO totally suppressed the differentiation even at a very low iron dosage of 1 μg/mL. From the Figure 3A, PAA encapsulated SPIO at the iron dosage of 10 μg/mL indeed totally suppressed osteoclasts differentiation. However, at the iron dosage of 1 ug/mL, the number of osteoclasts in PAA encapsulated SPIO group was still about 1/3 of that in the control group. Therefore, it would be better to explain what the “totally suppressed” really meant.

**Response:** We appreciate the important suggestion. The statement was not accurate and had been revised to “obviously suppressed” in Page 15 line 12.

3. In Figure 3, the HA group was tested and showed some kind of inhibitive effect on the osteoclasts differentiation. But the concentration of the HA could not be found. In addition, compared with the HA group, SPIO@3HA, SPIO@5HA showed similar moderate inhibitory effect, far less than that of SPIO group at the iron dosage of 1 μg/mL. Individually, the HA had inhibitory effect, but when incorporated with the SPIO in one nanosystem, HA reduced the inhibitory effect of SPIO. So, there is a question: what are the advantages of the SPIO@HA nanosystem compared with the simple mixture of SPIO and HA at the same Fe/Ca mole ratio? It is possible that the simple mixture of SPIO and HA might win better synergistic effect to regulate the osteoclastogenesis and osteogenesis.

**Response:** We appreciate the comments. For better readability, the concentration of HA was added to the figure 3. SPIO@HA nanosystem has certain advantages when compared with the simple mixture of SPIO and HA, including: HA encapsulated SPIO nanosystem (SPIO@HA) possessed good *T_2_* relaxivity (Fig. 1B), making the composite traceable under MRI after intravenous injection in vivo, as shown in Fig. 4. Secondly, as nanoparticles have different metabolic patterns, injection certain amount of SPIO and HA nanoparticles in mixture may result in different pharmacokinetic behaviors and SPIO/HA ratios in bone. Injection SPIO@HA nanocomposites should not change their pharmacokinetic behaviors and composition ratios in bone, and may be helpful in the bone remodeling process. We will evaluate the effects in our future study.

4. The SPIO@15HA showed great dual regulation of osteoclastogenesis and osteogenesis. But how the nanoparticles interacted with the cells was unclear. Did the nanoparticles interact with the membrane receptors or function after entering the cytoplasm? Besides, the SPIO@15HA could be taken out of the medium at different time points and the content change of SPIO@15HA could be further studied, which would be helpful to understand the life of SPIO@15HA during the treatment.

**Response:** We appreciate the reviewer’s comments. According to Ralph Weissleder and colleagues’ work (Nature Materials volume 13, pages125–138(2014)), nanoparticles studies for imaging including iron oxide nanoparticles would be internalized by reticular endothelial system (RES) macrophages and monocytes given their naturally high endocytosis activity, and the bone marrow is one of the important members of RES. And Xingxing Shi and co-worker (International Journal of Nanomedicine 2018:13 1457–1470) approved that hydroxyapatite nanoparticles confined to lysosomes and vesicles of hWJ-MSCs through endocytosis. Moreover, our previous work (Biomaterials Volume 203, May 2019, Pages 23-30) has approved that iron oxide nanoparticles could promote macrophage autophagy and inflammatory response through activation of toll-like Receptor-4 signaling. As a result, we speculate that the SPIO@15HA enters the cell by endocytosis and more specific mechanism to the point needs a further study.

Minor comments:

1. In Figure 2E and S1B, please point out the concentrations of different SPIO@HA nanocomposites in the stability study.

**Response:** The stability assessment of HA and different SPIO@HA was performed in the same hydroxyapatite concentration of 20 mg/mL. The stability assessment of PAA-coated SPIO was performed in the iron concentration of 0.5 mg/mL. The corresponding concentration of each nanoparticle was added in the description of Fig. 2E and Fig. S1B as follows: “HA, SPIO@3HA, SPIO@5HA and SPIO@15HA at hydroxyapatite concentration of 20 mg/mL, SPIO at iron concentration of 0.5 mg/mL” in the text.

2. In Page 15, line 22 to 24, the Fig. 1C in the sentence “Among them, SPIO@15HA exhibited the most obvious inhibitory effect with the lowest internalization (Fig. S2D and Fig. 1C).” might be wrong referred.

**Response:** We appreciate the reviewer’s comment, and the description was modified as“Among them, SPIO@15HA exhibited the most obvious inhibitory effect with the lowest internalization (Fig. S2D).” in the text.

***# Reviewer 3:***

1. Please describe the degree of protection of OVX by the nanocomposite.

**Response:** We appreciate the reviewer’s comments and added the corresponding description in the revised manuscript (highlighted in yellow) as following: “The obtained nanocomposites significantly prevented the bone loss of ovariectomized (OVX) mice and increased bone mineral density (BMD) by 9.4%, exhibiting high bone accumulation in MRI evaluation and reasonable biosafety profile.”

2. Please describe why hydroxyapatite was chosen as nanocomposites’ coating.

**Response:** We appreciate the reviewer’s comment. The nanocomposites with SPIO as the shell and HA as the core (HA@SPIO) may also be a good choice for the application of osteoblasts and osteoclasts for dual-regulation. However, due to the acid degradation of HA, it is difficult to prepare HA@SPIO nanocomposites. In the preparation of HA@SPIO nanocomposites, PAA-coated HA was synthesized firstly. To obtain SPIO shell, FeCl_2_ and FeCl_3_ were then added to the solution of PAA-coated HA and binds to the carboxyl group of PAA before the addition of alkaline solution. FeCl_2_ and FeCl_3_ are highly acidic, which leads to the degradation of HA. Therefore, SPIO@HA nanocomposites were chosen in this study thanks to their more convenient preparation method. In the section of introduction, the sentence of “SPIO were utilized as the core with the hydroxyapatite coating (SPIO@HA)” was changed to “SPIO@HA nanocomposites were prepared by coating HA on PAA-coated SPIO in a relatively simple method”.

3. Please state whether there are studies on SPIO-based inorganic nanomaterials for the treatment of osteoporosis, and related discussion.

**Response:** We really appreciate the valuable comments. The corresponding changes we made in revised manuscript were added to the section of introduction and highlighted in yellow as following: “According to the published works, iron oxide nanoparticles involved in osteoporosis treatment was mainly due to the iron supplementary or magnetic property. In our previous study, the clinically used superparamagnetic iron oxide nanoparticles (SPIO) were able to target bone defects and suppress osteoclastogenesis through TRAF6-p62-CYLD signaling complex regulation.”

4. How many mice were used in this study?

**Response:** There was a total of 24 C57BL/6 mice and 8 in each group used in the osteoporosis treatment. A few mice failed to survive after complicated experimental procedures due to poor surgical manipulations or vitality. As a result, the number of mice used in the osteoporosis treatment in each group was 7. For the MRI examination, a total of 2 SD rats and 1 in each group were used. For the pharmacokinetics evaluation, a total of 12 C57BL/6 mice and 4 in each group were used. The corresponding changes made in revised manuscript are highlighted in yellow.

*5.* The reason for choosing such an injection dose is not clear. SPIO@15HA (10 mg Fe/kg) and SPIO (10 mg Fe/kg)?

**Response:** In the in vitro experiment, SPIO@15HA was effective in inhibiting osteoclastic differentiation and promoting osteoblast differentiation at iron concentrations of 1 μg/mL and 10 μg/mL, respectively. However, when nanoparticles were injected into the body, their accumulation in bone marrow is much lower due to clearance by the RES system. According to our previous work (Acta Biomaterialia Volume 103, February 2020, Pages 281-292), clinically approved SPIO Ferumoxytol could suppress osteoclastogenesis and ovariectomy-induced bone loss at an injection dose of 10 mg/kg, which was chosen as a reference in this paper.

6. As mentioned that SPIO@HA nanocomposites have spindle-like morphology, but what is the rationale? Have authors tried to grow it into a different shell, such as a spherical shape?

**Response:** We appreciate the reviewer for the comment. Previous studies have shown that the reaction parameters such as pH, temperature, surface ligands and solvent systems could affect the size and morphology of HA (Chem Eng J 2014; 252: 173–184; Chem Mater 2005; 17: 5824-5830; Mat Sci Eng C-Mater 2016; 62: 160–165; Ceram Int 2003; 29: 629–633; Powder Technol 2010; 203: 315–321). In this work, HA and SPIO@HA nanocomposites with spindle-like morphology were obtained at 80 ℃, pH 10 after the addition of PAA or PAA-coated SPIO. The reaction temperature and pH were similar to the previous reported studies. Therefore, PAA may be the main driving force for the formation of spindle-like morphology HA. We want to explore the effect of SPIO@HA nanocomposites and their relative content in the nanocomposites on osteoblasts and osteoclasts in this study. In our further studies, SPIO@HA nanocomposites with different morphologies such as spherical, rod, needle and wire may be prepared by regulating the reaction conditions for the application of osteoblasts and osteoclasts dual-regulation.

7. Page 13 line 12, the unit of z-potential is incorrectly used.

**Response:** Thanks for the reviewer’s careful check for our mistake, the unit of z-potential was corrected as “mV” in the text.

8. What is the size distribution of class SPIO/HA.

**Response:** We appreciate the reviewer for pointing this out. The size distribution of SPIO, HA and SPIO@HA nanocomposites measured by DLS has been added and shown in Supporting Information (Figure S6). The corresponding changes made in revised manuscript are highlighted in yellow.

9. SPIO@HA inhibited BMM oriented osteoclast differentiation and promotes MSC oriented osteoblast differentiation. Figure 3. A and B, SPIO and SPIO@15HA shown similar results. Please describe more detail about the results. How was the histogram calculated in Figure 3.B?

**Response:** We appreciate the reviewer’s suggestion and has made some changes in the revised manuscript as the following: “Actin ring is an essential structure for bone resorption. The sealing zone formed by the rearrangement of actin in active osteoclasts appeared as a red circle under a fluorescence microscope and was counted as actin ring with the diameter greater than 20 μm. Same results were obtained in the actin ring staining assay, representing the suppression of osteoclasts resorption activity by nanoparticles.”

10. Figure 4. Accumulation of SPIO@15HA and SPIO nanoparticles in femoral bone marrow. The water control should show a bright signal under T2, but the control at 48 h in Figure 4. A SPIO@15HA group shown black. Please explain it.

**Response:** We appreciate the reviewer’s comments. Scanning at different TE in turn with the long TR is necessary for obtaining T_2_-map images by magnetic resonance, causing the whole scanning time is more than forty minutes for one rat, while the water control was placed unsteadily at 48h leading to accidentally moves during scanning. As a result, the water control signal is not collected in the image with TE value of 57 ms, showing a dark signal at that image. However, the control signal is successfully obtained in the image with the TE value of 68 ms, in which the water was still in the right place. So, we replaced all the images in Fig. 4A with the images obtaining at a TE value of 68 ms. Meanwhile, the corresponding description in “MR Imaging examination” was also modified in the revised manuscript and highlighted in yellow.

11. Please add reference to support your idea that bone resident macrophage could efficiently trap injected nanomaterial.

**Response:** We appreciate the reviewer’s valuable advice and have cited one related reference (Nature Materials volume 13, pages125–138(2014)) in the revised manuscript (Page 17 line 14).

12. Please describe Why choose OVX animal model to simulate osteoporosis?

**Response:** We appreciate the reviewer for pointing this out. The corresponding changes we made in revised manuscript are highlighted in yellow as following: “Ovariectomy usually causes a sharp decline of estrogen levels in mice, leading an imbalance between the activity of osteoblasts and osteoclasts, is a classic animal model to mimic osteoporosis in postmenopausal women.”

13. The study evaluated osteoporosis by factors such as trabecular number (Tb. N), trabecular thickness (Tb. Th) and connectivity density (Conn. D). Bone mineral density (BMD) is a clinical tool for diagnosing osteoporosis, predicting fracture risk and monitoring treatment. Can you provide BMD analysis in this study?

**Response:** We appreciate the reviewer’s comments. The BMD analysis has been added and shown in the revised manuscript (Fig. 6B). The corresponding changes we made are highlighted in yellow.

14. Signaling pathways related to osteoclastogenesis and osteogenesis were regulated by SPIO@15HA nanocomposites. GO analysis based on Gene Ontology and pathway analysis based on Kyoto Encyclopedia of Genes and Genomes (KEGG) was used to analysis the correlation among signal pathway. Do you design experiments to verify the signal path? Or please add some references to support your conclusion.

**Response:** We appreciate the reviewer’s comments. The gene expression profile microarray is like a massive PCR carried out in an integrated chip, where we got around 2000 differentially expressed genes (P<0.05, fold change>2) including the gene markers related to osteogenesis, such as BMP2, BMP6 and OPG. According to GO and KEGG analysis, the related pathways and biological processes were listed, in which signal pathway like TGF-β, PI3K-AKT and calcium signaling were well studied by previous literatures and proved related to osteogenic differentiation (Nat Rev Mol Cell Biol 2012;13:616-630; Biomaterials 2013;34:4404-17; Biomaterials 2015;36:44-54; Jak-Stat 2013;2:e23930; Acta Biomaterialia 59 (2017) 338–350). Studying signal pathways would be more helpful in understanding the mechanism SPIO@15HA in osteoporosis treatment. The downstream signaling of above-mentioned pathways will be investigated in our further studies.
